# Cancer anorexia‐cachexia syndrome is characterized by more than one inflammatory pathway

**DOI:** 10.1002/jcsm.13430

**Published:** 2024-03-13

**Authors:** Bruno Gagnon, Jessica Murphy, David Simonyan, Claudia A. Penafuerte, Jacinthe Sirois, Martin Chasen, Michel L. Tremblay

**Affiliations:** ^1^ Département de médecine familiale et de médecine d'urgence, Centre de recherche sur le cancer Université Laval, Centre de recherche du CHU de Québec Québec Canada; ^2^ Division of Clinical Epidemiology McGill University Health Centre Montreal Canada; ^3^ Department of Health, Kinesiology, and Applied Physiology Concordia University Montreal Canada; ^4^ Clinical and Evaluative Research Platform Université Laval, Centre de recherche du CHU de Québec Québec Canada; ^5^ Cura Therapeutics NEOMED Institute Saint‐Laurent Canada; ^6^ Rosalind and Morris Goodman Cancer Institute McGill University Montreal Canada; ^7^ Departments of Medicine and Family and Community Medicine University of Toronto Toronto Canada; ^8^ Department of Family Medicine McMaster University Hamilton Canada

**Keywords:** Anorexia, Cachexia, Cancer, Cytokines, Inflammation, Survival analysis

## Abstract

**Background:**

The interdependence of cytokines and appetite‐modifying hormones implicated in cancer anorexia‐cachexia syndrome (CACS) remains unclear. This study aimed to regroup these cytokines and hormones into distinct inflammatory (or non‐inflammatory) pathways and determine whether these pathways can classify patients with CACS phenotypes.

**Methods:**

Clinical characteristics of 133 patients [61.7% male; mean age = 63.4 (SD: 13.1) years] with advanced cancer prior to oncology treatments were documented, including weight loss history. Patients completed the Functional Assessment of Anorexia‐Cachexia Therapy (FAACT) questionnaire and Timed Up and Go test and had their sex‐standardized skeletal muscle index (z‐SMI) and fat mass index (z‐FMI) derived using computed tomography scans. Their plasma levels of cytokines and appetite‐modifying hormones were also determined. Date of death was recorded. Exploratory factor analysis (EFA) was used to regroup 15 cytokines and hormone into distinct inflammatory pathways (factors). For each patient, regression factor scores (RFS), which tell how strongly the patient associates with each factor, were derived. Two‐step cluster analysis on the RFS was used to classify patients into groups. CACS phenotypes were correlated with RFS and compared between groups. Groups' survival was estimated using Kaplan–Meier analysis.

**Results:**

Patients had low z‐SMI (mean = −3.78 cm^2^/m^2^; SD: 8.88) and z‐FMI (mean = 0.08 kg^2^/m^2^; SD: 56.25), and 62 (46.6%) had cachexia. EFA identified three factors: (F‐1) IFN‐γ, IL‐1β, Il‐4, IL‐6, IL‐10, IL‐12, TGFβ1 (positive contribution), and IL‐18 (negative); (F‐2) IL‐8, IL‐18, MCP‐1, TGFβ1, TNF‐α (positive), and ghrelin (negative); and (F‐3) TRAIL and leptin (positive), and TGFβ1 and adiponectin (negative). RFS‐1 was associated with cachexia (*P* = 0.002); RFS‐2, with higher CRP (*P* < 0.0001) and decreased physical function (*P* = 0.01); and RFS‐3 with better appetite (*P* = 0.04), lower CRP (*P* = 0.002), higher z‐SMI (*P* = 0.04) and z‐FMI (*P* < 0.0001), and less cachexia characteristics (all *P* < 0.001). Four patient groups were identified with specific RFS clusters aligning with the CACS continuum from no cachexia to pre‐cachexia, cachexia, and terminal cachexia. Compared to the other two groups, groups 1 and 2 had higher plasma levels of IL‐18 and TRAIL. Group 1 also had lower inflammatory cytokines, adiponectin, and CRP compared to the other three groups. Group 3 had inflammatory cytokine levels similar to group 2, except for TNF‐α and leptin which were lower. Group 4 had very high inflammatory cytokines, adiponectin, and CRP compared to the other 3 groups (all *P* < 0.0001). Groups 3 and 4 had worse cachexia characteristics (*P* < 0.05) and shorter survival (log rank: *P* = 0.0009) than the other two groups.

**Conclusions:**

This exploratory study identified three distinct pathways of inflammation, or lack thereof, characterizing different CACS phenotypes.

## Introduction

To clarify the role of inflammation and hormonal changes in tumour progression and cancer anorexia‐cachexia syndrome (CACS), blood levels of cytokines and appetite‐modifying hormones have been extensively investigated.^1^, ^2^ These biomarkers are highly interrelated, rendering their specific contribution to underlying pathophysiological processes difficult to estimate. Testing correlations between biomarkers can provide some information about the pathophysiological processes associated with CACS and survival, but it can fall short in describing commonalities^3^ and even lead to wrong interpretations. Therefore, a new method of investigating common pathways of chronic inflammation and hormonal changes in CACS is warranted. The primary objective of this study was to identify distinct inflammatory pathways in patients with advanced cancer based on plasma levels of cytokines and appetite‐modifying hormones. This study also explored if these pathways are associated with specific CACS characteristics.

## Methods

### Population

The population consisted of adult patients with a new diagnosis or recurrence of advanced cancer before any oncology treatment. Participants were recruited from the McGill University Health Centre and the Jewish General Hospital in Montreal, Canada between 19 February 2009 and 22 June 2011 for a prospective, longitudinal study on cancer cachexia. Date of death was recorded during the study. Inclusion criteria were an Eastern Cooperative Oncology Group performance status between 0 and 3, an expected life expectancy of at least 3 months, and no symptomatic brain metastases. Ethical approval was obtained from the McGill University Research Ethics Board, and all patients provided written informed consent.

### Baseline clinical assessment

Demographic and clinical characteristics were obtained by reviewing patients' medical charts, pathology reports, and imaging reports. Participants were interviewed about their weight history and had their weight and height measured. Participants who were unsure if they lost weight in the preceding 6 months were considered weight stable (<2% weight change) since patients and/or family are usually aware of weight changes. Body mass index (BMI) was calculated as weight (kg) per squared height (m^2^). Participants' appetite was assessed using the Functional Assessment of Anorexia‐Cachexia Therapy (FAACT) questionnaire [S1] and their physical function was evaluated using the Timed Up and Go (TUG) test [S2]. If participants agreed, their blood was collected in the morning in the non‐fasting state, and serum C‐reactive protein (CRP) (normal range = 0.0–5.0 mg/L) was analysed by the McGill University Health Centre and Jewish General Hospital central laboratories.

### Cytokine and hormone measurements

Plasma concentrations of cytokines and appetite‐modifying hormones including interleukin (IL)‐1α, IL‐1β, IL‐3, IL‐4, IL‐5, IL‐6, IL‐8, IL‐10, IL‐12 (p70), IL‐15, IL‐18, interferon (IFN)‐γ, tumour necrosis factor (TNF)‐α, transforming growth factor (TGF)‐β1, monocyte chemoattractant protein (MCP)‐1, TNF‐related apoptosis‐inducing ligand (TRAIL), leptin, ghrelin, and adiponectin, implicated in chronic inflammation and CACS, were measured as previously described^4^ (for measurement parameters, see Data S1).

### Assessment of CACS characteristics

Cancer‐associated weight loss (CAWL) was defined according to the grading system of Martin *et al*. that incorporates percent weight loss and body mass index.^5^ Participants' computed tomography (CT) scans performed for clinical reasons were used to quantify their muscle and fat cross‐sectional area at the third lumbar vertebrae (L3) by automated segmentation with MATLAB software (The MathWorks, Inc.; Natick, MA, USA) and manual correction by an expert in musculoskeletal anatomy (the MATLAB program was kindly provided by Dr. Vickie Baracos and Dr. Dana Cobzas, University of Alberta, Edmonton, AB, Canada). In patients with cancer, muscle and fat at the L3 landmark have been shown to correlate with appendicular skeletal muscle mass and whole‐body fat mass, respectively.^6^ Skeletal muscle index (SMI) and fat mass index (FMI) were calculated as muscle and fat cross‐sectional area (cm^2^) per squared height (m^2^). Participants were considered sarcopenic at baseline based on defined L3 SMI cut‐points: < 55 cm^2^/m^2^ for men and < 39 cm^2^/m^2^ for women.^6^ Cachexia was defined as per the International Consensus of Fearon *et al*.^7^: a weight loss > 5% in the last six months, or a weight loss of 2–5% in the last six months with a BMI < 20 kg/m^2^ and/or sarcopenia.

### Statistical analyses

The FAACT 5‐item subscale as defined by Gellhorn *et al*.^8^ was used to test for anorexia. TUG results were standardized using normative reference values based on sex and age.^9^ SMI was standardized for sex using sarcopenia cut‐points.^6^ FMI was converted from cm^2^/m^2^ to kg/m^2^ and standardized for sex.^10^ In at least 25% of cases, the plasma concentrations of IL‐1α, IL‐3 and IL‐5 were outside the limits of detections. These cytokines were excluded from further analysis. Spearman correlation coefficients (ρ) were estimated for all cytokines and hormones.

Exploratory factor analysis^11^ was conducted with the 12 remaining cytokines and hormones. Factor analysis can identify underlying structures, ‘factors’, which regroup highly correlated variables, in this case, biomarkers of inflammation and appetite. This method is highly recommended when a sound theoretical framework is hypothesized, as is the case here. Factor analysis is based on the variabilities of measured variables. While a correlation matrix estimates the relationships between two variables, factor analysis summarizes multiple measured variables with similar variabilities creating latent variables, factors. Factors are vectors which, by definition, are in a positive direction. Each variable included has its own variability along its own vector, either in the same direction as the factor, a positive loading, or in the opposite direction, a negative loading. The value of each variable loading is the ‘projection’ of the variable vector on the factor vector. This statistical procedure has the advantage of simplifying further analyses because less variables are needed in the model. It also prevents collinearity that can arise when variables are highly correlated. Exploratory factor analysis was conducted using unweighted least squares estimation method with oblimin rotation (δ = 0.5). Three factors with eigenvalues >1 were retained according to the Kaiser rule. Only factor loadings ≥ 0.300 are reported.

Regression factor scores (RFS) are a set of values that represent the degree to which each subject in a dataset is associated with a specific factor extracted from a factor analysis.^12^ RFS are calculated by regressing the observed variables (cytokine and hormones) on the factor scores estimated from the factor analysis. This regression method provides an estimated value (RFS) of each factor for each subject. To properly interpret the relationship between the RFS and the directional value of each biomarker within each factor, it's important to note that RFS scores have been normalized to have a mean of 0 and a standard deviation of 1. This means that biomarkers with positive values in a factor have a positive correlation with the RFS, regardless of whether the RFS score is positive or negative. Conversely, biomarkers with negative values have a negative correlation with the RFS, regardless of RFS value. The clinical relevance of these factors can be determined by correlating the RFS with subject characteristics of interest, in this case, CACS phenotypes.

Patients were classified into RFS clusters (groups) using two‐step cluster analysis based on similarities in their regression factor scores. Spearman correlations and Kruskal–Wallis tests were used to test the associations between RFS and cachexia characteristics. Multivariate backward linear, logistic, or cumulative ordinal regression were used as appropriate to test the effects of patient groups on CACS characteristics adjusted for confounders.

Kaplan–Meier survival analyses with log‐rank tests were conducted by patient groups and other prognostic predictors. Clusters analysis was performed using IBM SPSS Statistics for Windows, Version 25.0. (IBM Corp; Armonk, NY, USA). All other statistical analyses were performed using SAS version 9.4 (SAS Institute; Cary, NC, USA) with a two‐sided significance level set at *P* < 0.05. Kaplan–Meier graphs were produced using XLSTAT 2023 (Addinsoft; Paris, France).

## Results

Table 1 presents the demographic and CACS‐related characteristics of the patient population. The study included 133 patients, of whom 82 (61.7%) were male. Patients had a mean age of 63.4 (± 13.1) years and a variety of cancer types. The most common cancer types were pancreatic (27.1%) and ear‐nose‐throat (15.0%), and 80.8% of patients had stage IV cancer. Around half of the participants had cachexia, and the CACS‐related characteristics were well‐distributed.

**Table 1:** Patient characteristics

Sex (*n* = 133), *n* (%)	
Female	51 (38.3)
Male	82 (61.7)
Age, years (*n* = 133), mean (SD)	63.4 (13.1)
Cancer site (*n* = 133), *n* (%)	
Breast	13 (9.8)
Colorectal	18 (13.5)
Ear‐nose‐throat	20 (15.0)
Hepatobiliary	14 (10.5)
Non‐small cell lung	15 (11.3)
Pancreatic	36 (27.1)
Prostate	6 (4.5)
Upper gastro‐intestinal	11 (8.3)
Cancer stage (*n* = 130), *n* (%)	
III	25 (19.2)
IV	105 (80.8)
FAACT 5‐items,^a^ mean (SD) (*n* = 123)	6.44 (5.16)
6‐month recall weight loss (*n* = 133), *n* (%)	
<2%	59 (44.4)
2–5%	16 (12.0)
≥5%	58 (43.6)
Body mass index (*n* = 123), *n* (%)	
<20.0 kg/m^2^	27 (22.0)
20.0–24.9 kg/m^2^	47 (38.2)
25.0–29.9 kg/m^2^	31 (25.2)
≥30.0 kg/m^2^	18 (14.6)
Cancer‐associated weight loss^b^ (*n* = 124), *n* (%)	
0	29 (23.4)
1	25 (20.2)
2	12 (9.7)
3	32 (25.8)
4	26 (21.0)
Cachexia^c^ (*n* = 133), *n* (%)	
No	71 (53.4)
Yes	62 (46.6)
z‐skeletal muscle index,^d^ cm^2^/m^2^ (*n* = 98), mean (SD)	‐3.78 (8.88)
z‐fat mass index,^e^ kg^2^/m^2^ (*n* = 98), mean (SD)	0.08 (56.25)
z‐timed up and go,^f^ s (*n* = 120), mean (SD)	−1.40 (2.92)
C‐reactive protein, mg/L (*n* = 133), mean (SD)	18.22 (25.67)
Modified Glasgow prognostic score (*n* = 124), *n* (%)	
0	72 (58.1)
1	15 (12.1)
2	37 (29.8)

^a^ FAACT, Functional Assessment of Anorexia‐Cachexia Therapy questionnaire. The scores of the two following questions were reversed to go in the same direction as the other questions: ‘I have good appetite’ and ‘The amount I eat is sufficient’ (higher score meaning worse anorexia‐cachexia).^8^

^b^ Defined according to the grading system of Martin *et al*. that incorporates percent weight loss and body mass index. A higher grade is worse.^5^

^c^ Defined according to the International Consensus Statement: 6‐month recall weight loss > 5%; or a 6‐month recall weight loss 2–5% plus sarcopenia (skeletal muscle index <55 cm^2^/m^2^ for men and <39 cm^2/^m^2^ for women) and/or a BMI < 20 kg/m^2^.^7^

^d^ z‐skeletal muscle index: sex‐standardized skeletal mass index based on cut‐points of <55 cm^2^/m^2^ for men and <39 cm^2/^m^2^ for women.^6^

^e^ z‐fat mass index: sex‐standardized fat mass index.^10^

^f^ z‐timed up and go: sex‐ and age‐standardized timed up and go.^9^

Table 2 shows that there were moderate to strong positive correlations between the pro‐inflammatory cytokines, IFN‐γ, IL‐1β, IL‐6, IL‐8, IL‐10, IL‐12, MCP‐1, and TGF‐β1 (*r* = 0.44 to 0.94, *P* < 0.001), which, except for MCP‐1, correlated with TRAIL (*r* = −0.20 to −0.42, from *P* < 0.05 to *P* < 0.001). Except for IL‐8 and MCP‐1, the pro‐inflammatory cytokines also correlated negatively with IL‐18 (*r* = −0.29 to −0.38, *P* < 0.01). TNF‐α correlated positively with IL‐8, IL‐18, MCP‐1, and TGF‐β1 (*r* = 0.20 to 0.28, *P* < 0.05 to *P* < 0.01). Leptin correlated positively with TRAIL (*r* = 0.25, *P* < 0.01) and negatively with IL‐8 and adiponectin (*r* = −0.22, *P* < 0.05), while ghrelin correlated negatively with TGF‐β1 and IL‐1β (*r* = −0.20 to −0.34, *P* < 0.05 to *P* < 0.001).

**Table 2:** Spearman correlation matrix of cytokine and hormone plasma levels

	MCP‐1	IFN‐γ	IL‐1β	IL‐4	IL‐6	IL‐8	IL‐10	IL‐12	IL‐18	TGF‐β1	TRAIL	Ghrelin	Leptin	TNF‐α	Adiponectin
MCP‐1	1.00														
IFN‐γ	0.52^d^	1.00													
IL‐1β	0.51^d^	0.93^d^	1.00												
IL‐4	0.53^d^	0.94^d^	0.95^d^	1.00											
IL‐6	0.57^d^	0.91^d^	0.86^d^	0.88^d^	1.00										
IL‐8	0.64^d^	0.71^d^	0.70^d^	0.68^d^	0.70^d^	1.00									
IL‐10	0.48^d^	0.85^d^	0.77^d^	0.80^d^	0.85^d^	0.61^d^	1.00								
IL‐12	0.44^d^	0.77^d^	0.69^d^	0.68^d^	0.74^d^	0.65^d^	0.87^d^	1.00							
IL‐18	−0.01	−0.38^d^	−0.38^d^	−0.42^d^	−0.35^d^	−0.08	−0.34^d^	−0.32^d^	1.00						
TGF‐β1	0.45^d^	0.71^d^	0.71^d^	0.71^d^	0.70^d^	0.56^d^	0.57^d^	0.44^d^	−0.29^b^	1.00					
TRAIL	−0.12	−0.30^d^	−0.21^a^	−0.28^b^	−0.40^d^	−0.30^d^	−0.28^b^	−0.20^a^	0.09	−0.30^d^	1.00				
Ghrelin	−0.15	−0.15	−0.20^a^	−0.20^a^	−0.16	−0.16	−0.11	0.01	−0.02	−0.34^d^	0.14	1.00			
Leptin	−0.11	−0.16	−0.16	−0.17	−0.14	−0.22^a^	−0.10	−0.09	0.02	−0.15	0.25^b^	−0.10	1.00		
TNF‐α	0.30^b^	0.16	0.15	0.12	0.17	0.27^b^	0.03	0.06	0.28^b^	0.20^a^	−0.10	−0.13	0.01	1.00	
Adiponectin	−0.11	0.04	0.02	−0.004	0.08	0.11	0.06	0.07	0.04	0.07	−0.14	−0.03	−0.22^a^	0.13	1.00
RFS‐1	0.46^d^	0.94^d^	0.92^d^	0.92^d^	0.90^d^	0.65^d^	0.88^d^	0.81^d^	−0.56^d^	0.71^d^	−0.29^d^	−0.16	−0.11	0.05	−0.02
RFS‐2	0.74^d^	0.42^d^	0.43^d^	0.40^d^	0.46^d^	0.65^d^	0.32^d^	0.30^d^	0.32^d^	0.50^d^	−0.13	−0.35^d^	0.04	0.68^d^	0.02
RFS‐3	−0.14	−0.28^c^	−0.25^c^	−0.27^c^	−0.36^d^	−0.35^d^	−0.24^c^	−0.18^a^	0.15	−0.41^d^	0.58^d^	−0.00	0.75^d^	−0.00	−0.59^d^

RFS, regression factor score for each factor (1, 2, and 3).

^a^ *P* < 0.05.

^b^ *P* < 0.01.

^c^ *P* < 0.005.

^d^ *P* < 0.0001.

Table 3(A) presents the results of the factor analysis. Three factors were identified explaining 55.2% of the variance with small correlations between factors (*r*_I‐II_ = 0.209; *r*_I‐III_ = −0.190; *r*_II‐III_ = −0.081). Factor 1 includes all the pro‐inflammatory cytokines (IL‐1β, IL‐4, IL‐6, IL‐10, IL‐12, and TGF‐β1) with positive loadings, and IL‐18 with a negative loading. Factor 2 includes TNF‐α, IL‐8, IL‐18, MCP‐1, and TGF‐β1 with positive loadings, and ghrelin with a negative loading. Factor 3 includes TRAIL and leptin with positive loadings, and TGF‐β1 and adiponectin with negative loadings.

**Table 3:** Cytokine and hormone factor loading patterns, regression factor scores, and factor score cluster profiles (*n* = 133)

(A)			
	Factor 1	Factor 2	Factor 3
Factor loadings^a^ ^,^ ^b^ for cytokines and hormones			
IFN‐γ	0.890	‐	‐
IL‐1β	0.747	‐	‐
IL‐4	0.896	‐	‐
IL‐6	0.588	‐	‐
IL‐8	‐	0.637	‐
IL‐10	0.807	‐	‐
IL‐12	0.801	‐	‐
IL‐18	−0.556	0.523	‐
MCP‐1	‐	0.739	‐
TGF‐β1	0.412	0.359	−0.349
TNF‐α	‐	0.670	‐
TRAIL	‐	‐	0.627
Leptin	‐	‐	0.767
Ghrelin	‐	−0.318	‐
Adiponectin	‐	‐	−0.593
Eigenvalue	5.063	1.833	1.381
Explained variance,%: Total (55.2%)	33.75	12.22	9.21
Factor correlations: *r*_I‐II_ = 0.209; *r*_I‐III_ = −0.190; *r*_II‐III_ = −0.081			
Regression‐factor‐scores,^c^ median (IQR)	0.17 (−0.36, 0.75)	0.05 (−0.63, 0.61)	0.06 (−0.57, 0.74)

(B)			
Patient grouping by regression‐factor‐score cluster profiles,^d^ median (IQR)			
Group 1: 20 patients (15.0%)	−1.45 (−2.49, −1.10)	−0.91 (−2.18, 0.77)	0.02 (−0.57, 0.82)
Group 2: 35 patients (26.3%)	0.06 (−0.37, 0.61)	0.38 (0.00, 0.70)	0.87 (0.73, 1.19)
Group 3: 39 patients (29.3%)	0.16 (−0.10, 0.46)	−0.64 (−0.92, −0.37)	−0.03 (−0.53, 0.43)
Group 4: 39 patients (29.3%)	0.83 (0.38, 1.04)	0.56 (0.28, 0.95)	−0.75 (−1.52, −0.06)

IQR, inter‐quartile range.

^a^ Unweighted least squares extraction procedure with direct *Oblimin rotation* (δ = 0.5). 11 iterations; *n* = 133.

^b^ Factor analysis was performed using log‐transformed cytokines and appetite‐modifying hormones. Only pattern loadings ≥0.300 are reported.

^c^ Scores are normalized with mean = 0 and SD = 1.

^d^ Two‐step cluster analysis performed on regression factor scores.

**Figure 1:**
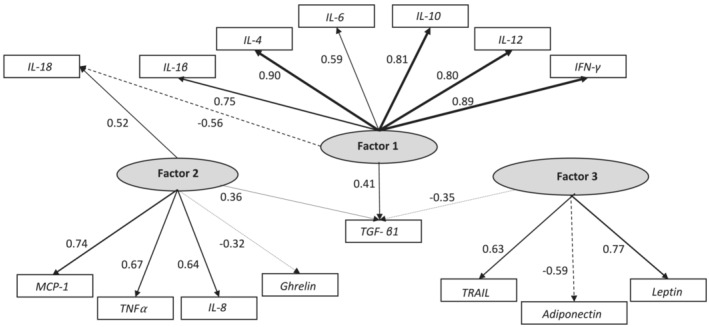
Path diagram of factor analysis of cytokines and appetite‐modifying hormones.

Figure 1 shows a schematic view of the relationships between the cytokines/hormones and each factor, facilitating the understanding of how factor analysis regrouped multiple variables into three factors or latent variables. Regression Factor Score (RFS) descriptive values for each of the three factors (RFS‐1, RFS‐2, and RFS‐3) are also presented at the bottom of Table 3(A) (see their Spearman correlations with cytokines and hormones in Table 2). For example, the Factor 1 RFS (RFS‐1) (median: 0.17, IQR:‐0.36, 0.75) provides an overall score which takes into account the positive values of IFN‐γ, IL‐1β, IL‐4, and TGF‐β1, and negative value of IL‐18.

Table 3(B) presents the median (interquartile range [IQR]) RFS for the groups with specific RFS cluster profiles. Group 1 (*n* = 20) was characterized by a negative RFS‐1 and ‐2 and a positive RFS‐3; group 2 (*n* = 35) by a positive RFS‐1, −2, and −3; group 3 (*n* = 39) by a positive RFS‐1 and a negative RFS‐2 and ‐3; and group 4 (*n* = 39) by a positive RFS‐1 and ‐2 and a negative RFS‐3.

Table 4(A) presents the correlations between RFS and CACS characteristics measured on a continuous scale. RFS‐1 did not correlate with FAACT 5‐item, TUG, FMI, or SMI, but correlated weakly with CRP (*r* = 0.17, *P* = 0.04). RFS‐2 correlated positively with TUG (the higher the RFS, the longer the time to perform the TUG, *r* = 0.22; *P* = 0.01), and CRP (*r* = 0.35, *P* < 0.0001). RFS‐3 correlated negatively with FAACT 5‐item (the higher the RFS, the better the appetite, *r* = −0.19, *P* = 0.04) and CRP (*r* = −0.27, *P* = 0.002), and positively with z‐FMI (*r* = 0.61, *P* < 0.0001) and z‐SMI (*r* = 0.21, *P* = 0.04). Table 4(B) presents the median (IQR) of the RFS by CACS categorical characteristics. A higher median RFS‐1 was significantly associated with cachexia (*P*  0.002) and more 6‐month recall weight loss (*P* = 0.003). A higher median RFS‐2 was significantly associated with the male sex (*P* = 0.05) and worse modified Glasgow prognostic score (mGPS) (*P* < 0.0001). On the other hand, a higher median RFS‐3 was significantly associated with no cachexia (*P* < 0.0001), higher BMI (*P* < 0.0001), better mGPS (*P* = 0.02), less 6‐month recall weight loss (*P* < 0.0001), and better CAWL (*P* < 0.0001), and was not associated with sex.

**Table 4:** Relationships between cancer anorexia‐cachexia syndrome characteristics and regression factor scores

(A) Spearman correlation coefficients						
Varable (*N*)	RFS‐1, ρ	*P*‐value	RFS‐2, ρ	*P*‐value	RFS‐3, ρ	*P*‐value
FAACT 5‐Items^a^ (123)	0.07	0.45	0.003	0.97	−0.19	0.04
*z*‐timed up and go^b^ (120)	0.13	0.16	0.22	0.01	−0.08	0.36
*z*‐fat mass index^c^ (98)	−0.03	0.77	0.10	0.33	0.61	<0.0001
*z*‐skeletal muscle index^d^ (98)	−0.03	0.77	−0.02	0.83	0.21	0.04
C‐reactive protein (133)	0.18	0.04	0.35	<0.0001	−0.27	0.002

(B) Regression factor scores by cancer anorexia‐cachexia syndrome characteristics						
Variable (*N*)	RFS‐1, Median (IQR)	*P*‐value*	RFS‐2, Median (IQR)	*P*‐value*	RFS‐3, Median (IQR)	*P*‐value*
Sex (133)		0.18		0.05		0.36
Female (51)	−0.001 (−0.368, 0.557)		−0.122 (−0.877, 0.546)		0.223 (−0.436, 0.786)	
Male (82)	0.283 (−0.300, 0.800)		0.249 (−0.461, 0.685)		0.042 (−0.713, 0.740)	
Cachexia^e^ (133)		0.002		0.15		<0.0001
No (71)	−0.001 (−0.748, 0.456)		−0.108 (−0.791, 0.546)		0.392 (−0.238, 0.921)	
Yes (62)	0.461 (−0.239, 0.869)		0.249 (−0.478, 0.657)		−0.234 (−1.052, 0.434)	
Body mass index (123)		0.98		0.13		<0.0001
< 20.0 kg/m^2^ (27)	0.157 (−0.364, 0.753)		−0.046 (−0.749, 0.702)		−0.526 (−1.672, −0.066)	
20.0‐24.9 kg/m^2^ (47)	0.216 (−0.367, 0.683)		−0.204 (−0.857, 0.484)		−0.042 (−0.577, 0.547)	
25.0‐29.9 kg/m^2^ (31)	0.162 (−0.553, 0.825)		0.280 (−0.374, 0.948)		0.210 (−0.060, 0.616)	
≥ 30.0 kg/m^2^ (18)	0.329 (−0.151, 0.765)		0.243 (−0.430, 0.796)		0.780 (0.434, 0.921)	
Modified Glasgow prognostic score (124)		0.06		<0.0001		0.02
0 (72)	0.167 (−0.489, 0.579)		−0.151 (−0.799, 0.414)		0.252 (−0.330, 0.864)	
1 (15)	−0.001 (−0.368, 0.677)		0.214 (−0.790, 0.973)		−0.042 (−0.803, 0.662)	
2 (37)	0.480 (−0.111, 0.900)		0.573 (0.280, 1.261)		−0.377 (−1.390, 0.513)	
6‐month recall weight loss (133)		0.003		0.38		<0.0001
<2% (59)	−0.052 (−0.679, 0.551)		−0.046 (−0.749, 0.484)		0.392 (−0.111, 0.937)	
2‐5 % (16)	0.061 (−1.694, 0.318)		−0.195 (−0.748, 0.705)		0.536 (−0.305, 0.805)	
>5% (58)	0.499 (−0.104, 0.880)		0.314 (−0.452, 0.685)		−0.353 (−1.086, 0.266)	
Cancer‐associated weight loss^f^ (124)		0.10		0.65		<0.0001
0 (29)	0.007 (−0.293, 0.611)		0.060 (−0.430, 0.463)		0.610 (−0.004, 0.921)	
1 (25)	−0.157 (−0.899, 0.435)		−0.436 (−1.094, 0.468)		0.455 (−0.073, 0.809)	
2 (12)	0.466 (−0.401, 0.825)		−0.005 (−0.638, 0.676)		0.177 (−0.547, 0.577)	
3 (32)	0.419 (0.094, 0.791)		0.263 (−0.685, 0.644)		−0.037 (−0.538, 0.503)	
4 (26)	0.137 (−0.364, 0.982)		0.074 (−0.637, 0.558)		−0.640 (−1.323, −0.218)	

IQR, interquartile range; RFS, regression factor score for each factor (1, 2 and 3).

^a^ FAACT 5‐items: Functional Assessment of Anorexia‐Cachexia Therapy questionnaire. The scores of two following questions were reversed to go in the same direction as the other questions: ‘I have good appetite’ and ‘The amount I eat is sufficient’ were reversed to go in the same direction as the other questions (higher score meaning worse anorexia‐cachexia) [8].

^b^ *z*‐timed up and go: sex‐ and age‐standardized timed up and go [9].

^c^ *z*‐fat mass index: sex‐standardized fat mass index [10].

^d^ *z*‐skeletal muscle Index: sex‐standardized skeletal muscle index based on cut‐points of 55 cm^2^/m^2^ for men and <39 cm^2/^m^2^ for women [6].

^e^ Defined according to the International Consensus Statement: 6‐month recall weight loss >5%; or a 6‐month recall weight loss 2–5% plus sarcopenia and/or a BMI ≤ 20 kg/m^2^ [10].

^f^ Defined according to the grading system of Martin et al. that incorporates percent weight loss and body mass index. A higher grade is worse [7].

* Kruskal–Wallis test.

Table 5(A) presents differences in cytokine and hormone plasma levels and regression factor scores between the 4 patient groups. Group 1 was characterized by high IL‐18, TRAIL, leptin and ghrelin, and low inflammatory cytokines (IL‐1β, IL‐4, IL‐6, IL‐8, IL‐10, IL‐12, INF‐γ), and low TGF‐β1, TNF‐α, and MCP‐1. Compared to group 1, group 2 had slightly increased inflammatory cytokines, low MCP‐1, high TNF‐α, very high leptin, and similar levels of IL‐18 and TRAIL. Group 3 was very similar to group 2, but had lower IL‐18, TNF‐α, TRAIL and leptin, and higher ghrelin. Group 4 had very high adiponectin and inflammatory cytokines including TGF‐β1 and TNF‐α, and low IL‐18, TRAIL, ghrelin, and leptin. Overall, inflammatory cytokine plasma levels increased from group 1 to 4, while IL‐18 and TRAIL were higher in groups 1 and 2 than in groups 3 and 4. Of special interest, CRP was extremely high in group 4. Table 5(B) presents a schematic way to describe some relevant cytokines and leptin plasma levels facilitating the characterization of each patient. For example, it is easy to see that patients in groups 1 and 2 are different from those in group 3 as they have higher IL‐18 and TRAIL. Patients in group 2 were the only group with high leptin. Similar comparisons can be made between other groups.

**Table 5:** Cytokine and hormone plasma levels and regression factor scores by groups of patients and an example using cytokines to classify patients

(A) Cytokine and hormone plasma levels and regression factor scores by groups of patients						
Variable	Overall (*n* = 133)	Group 1 (*n* = 20)	Group 2 (*n* = 35)	Group 3 (*n* = 39)	Group 4 (*n* = 39)	*P*‐value*
	Median (IQR) (range)	Median (IQR)	Median (IQR)	Median (IQR)	Median (IQR)	
IL‐1β (pg/mL)	3.44 (1.18, 9.23) (0.10, 35.85)	0.10 (0.10. 0.40)	3.25 (1.39, 7.32)	2.12 (1.39, 5.28)	13.55 (7.47, 18.45)	<0.0001
IL‐4 (pg/mL)	5.91 (3.01, 10.57) (0.10, 31.42)	1.20 (0.27, 2.45)	5.83 (3.01, 8.24)	4.77 (3.28, 7.52)	14.81 (9.19, 18.78)	<0.0001
IL‐6 (pg/mL)	19.38 (9.28, 41.46) (0.10) (299.88)	4.94 (2.72, 7.16)	13.97 (8.62, 30.94)	17.71 (10.00, 28.44)	56.04 (32.59, 87.66)	<0.00001
IL‐8 (pg/mL)	27.22 (5.61) (61.25) (0.01, 2409.00	0.87 (0.10, 15.69)	19.23 (6.51, 62.93)	14.03 (3.49, 28.69)	78.75 (41.64, 124.37)	<0.0001
IL‐10 (pg/mL)	12.28 (5.02, 34.73) (0.10, 473.89)	1.71 (0.42, 4.27)	10.05 (5.77, 16.41)	11.31 (4.98, 20.26)	37.64 (25.19, 54.86)	<0.0001
IL‐12 (pg/mL)	21.11 (5.17, 58.66) (0.10, 995.72)	0.10 (0.10, 1.38)	21.11 (7.20, 47.83)	20.06 (9.21, 32.59)	58.66 (32.61, 92.83)	<0.0001
IL‐18 (pg/mL)	86.44 (56.83, 128.08) (1.70, 298.55)	113.70 (89.12, 201.90)	110.88 (73.70, 148.28)	73.67 (58.88. 99.40)	79.19 (58.32, 159.33)	0.0015
IFN‐γ (pg/mL)	226.21 (86.51, 478.03) (0.10, 478.03)	14.06 (0.10, 39.56)	182.30 (103.70, 366.39)	170.92 (114.22. 278.26)	537.82 (318.18, 789.73)	<0.0001
MCP‐1 (pg/mL)	46.09 (34.67, 69.05) (14.44, 339.70)	33.09 (26.41, 67.07)	47.81 (41.08, 58.81)	39.22(30.73, 44.19)	69.31 (55.90, 83.70)	<0.0001
TGF‐β1 (ng/mL)	14.89 (9.24, 27.92) (3.49, 75.99)	7.25 (4.92, 10.49)	12.78 (9.34, 17.90)	13.73 (8.62, 19.44)	38.01 (17.68, 47.09)	<0.0001
TNF‐α (pg/mL)	4.17 (2.65, 5.89) (0.82, 29.36)	2.81 (1.48, 4.93)	5.54 (3.76, 7.14)	2.97 (2.23, 4.43)	4.82 (3.48, 7.42)	<0.0001
TRAIL (pg/mL)	86.44 (56.83, 128.08) (1.70, 298.55)	126.26 (61.64, 170.51)	120.78 (77.11, 147.51)	78.97 (63.39, 124.38)	64.16 (30.59, 101.51)	<0.0001
Adiponectin (μg/mL)	39.71 (25.25, 67.16) (2.24, 680.27)	40.17 (24.65, 99.49)	27.97 (19.29, 38.60)	42.19 (27.94, 52.77)	67.16 (31.99, 93.29)	0.0002
Ghrelin (pg/mL)	7.17 (5.03, 10.46) (0.10, 88.49)	7.93 (6.27, 18.35)	5.99 (4.04, 9.68)	8.77 (5.19, 15.58)	6.45 (3.25, 8.65)	0.0115
Leptin (ng/mL)	4.36 (1.52, 11.45) (0.01, 59.43)	4.57 (1.41, 7.31)	14.17 (7.84, 24.13)	3.08 (1.31, 6.42)	2.61 (0.63, 4.28)	<0.0001
CRP (mg/dL)	7.50 (2.60, 23.90) (0.30, 168.70)	6.05 (0.90, 16.55)	6.20 (2.32, 12.90)	6.82 (1.73, 18.60)	21.66 (4.60, 50.70)	0.0094
RFS‐1	0.17 (−0.36, 0,75) (−3.85, 1.45)	−1.45 (−2.49, −1.10)	0.06 (−0.37, 0.61)	0.16 (−0.10, 0.46)	0.83 (0.38, 1.04)	<0.0001
RFS‐2	0.05 (−0.63, 0,61) (−2.68, 2.67)	−0.91 (−2.18, 0.77)	0.38 (0.00, 0.70)	−0.64 (−0.92, −0.37)	0.56 (0.28, 0.95)	<0.0001
RFS‐3	0.06 (−0.57, 0,74) (−3.10, 2.26)	0.02 (−0.57, 0.82)	0.87 (0.73, 1.19)	−0.03 (−0.53, 0.43)	−0.75 (−1.52, −0.06)	<0.0001

(B) Example of a minimum set of cytokine plasma levels for classifying patients				
Cytokines	Group 1	Group 2	Group 3	Group 4
			(*n* = 39)	(*n* = 39)
IL‐6 (pg/mL)	<8	8–30	8–30	>30
IL‐18 (pg/mL)	>100	>100	<100	<100
TRAIL (pg/mL)	>110	>110	≤110	≤110
TNF‐α (pg/mL)	<4	>4	<4	>4
Leptin (ng/mL)	<7	>7	<7	<7
Other	To be tested if therapeutic target			

CRP, C‐reactive protein; IQR, interquartile range; RFS, regression factor score for each factor (1, 2, and 3).

* Kruskal–Wallis test.

Table 6 presents the final results of backward multivariate regressions of CACS phenotypes by patient groups. The initial models included cancersite, stage, sex, and age as predictors Predictors with *P* < 0.05 were subsequently removed in the backward regressions. When the estimates were very similar in two or three groups, they were merged to improve precision (see Data S1 for details of the analysis and effects of confounders). z‐SMI was significantly lower for patients in groups 3 and 4 (−3.56; 95% CI: −7.05, −0.08; *P* = 0.045) than those in the other two groups, and z‐FMI was significantly lower for patients in groups 1, 3, and 4 compared to those in group 2 (−60.24; 95% CI: −83.62, −36.87; *P* < 0.0001). CRP was significantly higher in groups 3 and 4 (14.33; 95% CI: 5.82, 22.84; *P* = 0.001) than groups 1 and 2. Patients in groups 3 and 4 were more likely to present with cachexia phenotypes than those in groups 1 and 2 (4.01; 95% CI: 1.84, 9.09; *P*  0.0006). Patients in group 4 were more likely to recall greater weight loss in the previous 6 months (4.23; 95% CI: 1.94, 9.78; *P* = 0.0003) than those in the other 3 groups. Patients in groups 1, 3, and 4 were more likely to have lower BMI than those in group 2 (5.54; 95% CI: 2.48, 12.84; *P* < 0.0001). Note that in cumulative ordinal regressions (6‐months recall weight loss and BMI), an odds ratio above 1 indicates greater odds of being in the subsequent categories. In multivariate regression analysis, the FAACT 5‐item did not differ between patient groups (*P* = 0.7), but was higher in women than men (2.2; 95% CI: 0.4, 4.0; *P* = 0.02), and in those with pancreatic cancer (5.7; 95% CI: 2.6, 87; *P* = 0.0004) and other cancer types (3.2; 95% CI: 0.4, 5.9; *P* = 0.03) than those with breast and prostate cancers.

**Table 6:** Backward multivariate regression analyses of patient groups on cachexia‐related outcomes

Outcomes	Predictors^a^			
*Linear regression*	Patient groups	*N* (%)	*Β* (95% CI)	*P*‐value
*z*‐skeletal muscle index^b^ ^,^ ^c^ (cm^2^/m^2^)	Group 3 & 4 Group 1 & 2	60 (61.22) 38 (38.78)	‐3.56 (‐7.05, ‐0.08) Reference	0.045
*z*‐fat mass index^d^ (kg/m^2^)	Group 1,3, & 4 Group 2	74 (75.51) 24 (24.49)	‐60.24 (‐83.62, ‐36.87) Reference	<0.0001
C‐reactive protein^e^ (mg/L)	Group 3 & 4 Group 1 & 2	78 (58.65) 55 (41.35)	14.33 (5.82, 22.84) Reference	0.001

*Logistic regression*	Patient groups	*N* (%)	OR (95% CI)	*P*‐value
Cachexia^e^ ^,^ ^f^ (yes vs. no)	Group 3 & 4 Group 1 & 2	78 (58.65) 55 (41.35)	4.01 (1.84, 9.09) Reference	0.0006

Cumulative ordinal regression	Patient groups	*N* (%)	OR (95% CI)	*P*‐value
6‐month recall weight loss (%) (<2; 2–5; ≥5)	Group 4 Group 1, 2, & 3	39 (29.32) 94 (70.68)	4.23 (1.94, 9.78) Reference	0.0003
Body mass index (kg/m^2^) ≥30.0; 25.0–29.9; 20.0–24.9; <20.0	Group 1, 3, & 4 Group 2	98 (73.68) 35 (26.32)	5.54 (2.48, 12.84) Reference	<0.0001

^a^ All initial models included group, sex, age, cancer site, and cancer stage. Predictors with *P* < 0.05 were subsequently removed in the backward regression. The results for the final model are presented.

^b^ *z*‐skeletal muscle index: sex‐standardized skeletal muscle index based on cut‐points of < 55 cm^2^/m^2^ for men and < 39 cm^2^/m^2^ for women [9].

^c^ Final model also included sex as a predictor.

^d^ *z*‐fat mass index: sex‐standardized fat mass index [13].

^e^ Final model also included cancer site as a predictor.

^f^ Defined according to the International Consensus Statement: 6‐month recall weight loss >5%, or a 6‐month recall weight loss 2–5% plus sarcopenia and/or a BMI < 20 kg/m^2^ [10].

**Figure 2:**
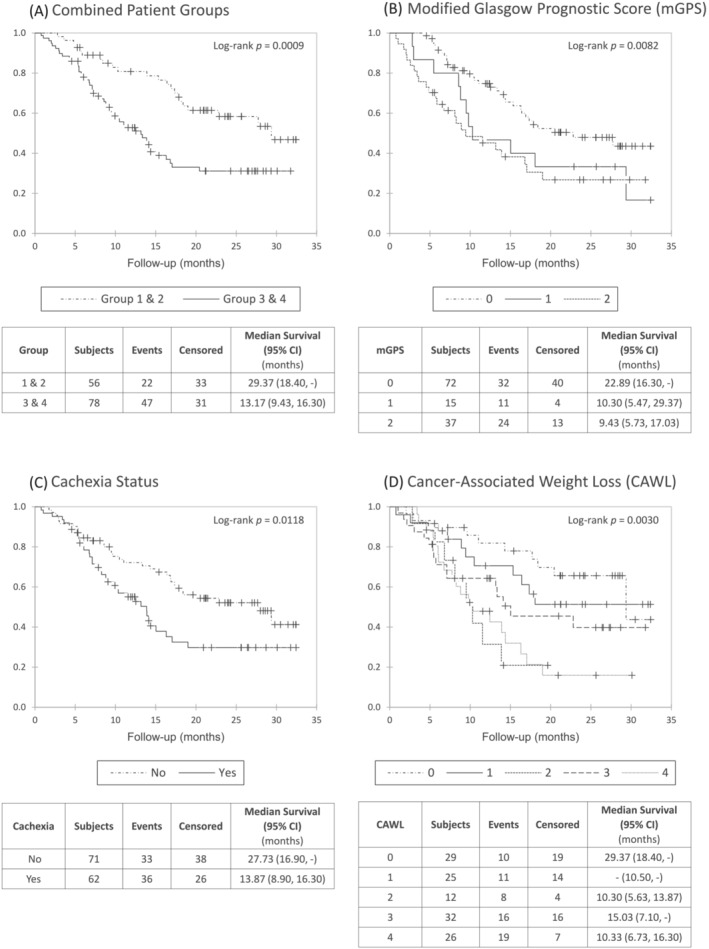
Kaplan–Meier curves according to prognostic score systems. ‘+’ censored.

Figure 2 presents Kaplan–Meier graphs. Because groups 1 and 2 (without anorexia‐cachexia), and groups 3 and 4 (with anorexia‐cachexia) had very similar survival estimates (see Data S1), they were combined. Groups 3 and 4 had worse survival than groups 1 and 2 [13.2 months (95% CI: 9.4, 16.3) and 29.4 months (95% CI: 18.4, −), respectively, log rank *P* = 0.0009] (a). The other Kaplan–Meier graphs present three other commonly used prognostic scores: mGPS (b), cachexia status (c), and CAWL (d). Of note, the patients' survival is better estimated by the prognostication using RFS clusters by combined patient groups (a) than the other prognostic systems (see log rank *P* values).

## Discussion

Unravelling the contributions of cytokines and appetite‐modifying hormones to the pathophysiology and phenotyping of CACS presents an ongoing challenge due to their complex interrelationships. The present study is the first to attempt to simplify this interpretation by examining inflammatory pathways and hormonal changes in cancer using factor analysis, regression factor scores, and cluster analysis. In patients with newly diagnosed advanced cancer before any oncological treatments, we identified three independent inflammatory (or non‐inflammatory) pathways, or *statistical biomarkers*. The three identified pathways are biologically plausible. The first comprises known inflammatory cytokines and can be rightly called an ‘inflammatory’ statistical biomarker. The second, due to the presence of high TNF‐α, can be called a ‘necrotic‐anorexic’ statistical biomarker. As this second biomarker also includes other specific cytokines such as IL‐8, IL‐18, and MCP‐1, it may represent a pathophysiological pathway distinct from the inflammatory one. The third has high TRAIL and leptin and can therefore be called a ‘non‐inflammatory/non‐anorexic’ statistical biomarker. A pivotal step in our analysis was the computation of RFS which summarize the weight of the contribution of all cytokines and hormones to each factor in a unique score for each patient. Therefore, each patient has a unique score, or RFS, for each statistical biomarker. Correlating RFS with CACS phenotypes lead to an improved understanding of the relationships between biomarkers and these phenotypes which supports the above nomenclature. Furthermore, because each patient was characterized by three statistical biomarkers, it was possible to define four groups of patients with similar RFS profiles. These four groups were found to be distinct in their CACS characteristics.

The ‘inflammatory’ statistical biomarker correlated with cachexia and weight loss and not with current fat and muscle mass indices, suggesting that inflammatory cytokines alone may not explain changes in body composition. IL‐10 contributed positively to the ‘inflammatory’ statistical biomarker suggesting it has a pro‐inflammatory role and not an anti‐inflammatory role^1^ in this population. TGF‐β1, which correlated mainly with the ‘inflammatory’ statistical biomarker, is known for its immunosuppressive properties. However, in the presence of pro‐inflammatory cytokines, it can induce the differentiation of IL‐17‐producing T cells, thereby promoting cancer progression.^13^ TGF‐β1, also part of the ‘necrotic‐anorexic’ statistical biomarker, can promote muscle wasting by increasing protein degradation and inhibiting protein synthesis.^14^ This, in turn, can lead to muscle weakness as previously demonstrated in a cohort of patients with cancer metastasized to bone.^15^ Our findings suggest that this relationship may be present in a wide variety of cancer types with or without bone metastasis.

Interestingly, IL‐18, a potent pro‐inflammatory cytokine, had a negative contribution to the ‘inflammatory’ statistical biomarker and a positive contribution to the ‘necrotic‐anorexic’ statistical biomarker. IL‐18 is normally expressed but to become biologically active, it needs to be proteolytically cleaved by caspase‐1 in a process that requires high IFN‐γ.^16^, ^17^ IL‐18 may therefore be ‘consumed’ in patients with higher ‘inflammatory’ statistical biomarkers, explaining its negative contribution. Furthermore, IL‐18 binding protein is upregulated in various cancers,^18^ and in response to uncontrolled inflammation, can bind and inactivate IL‐18.^19^, ^20^ IL‐18 has also been suggested to induce anorexia and weight loss when injected peripherally or centrally in mice.^21^ Our study does not support this hypothesis.

TNF‐α is a well‐studied pro‐inflammatory cytokine understood to induce anorexia peripherally and centrally by inhibiting the secretion of ghrelin, and to trigger muscle wasting through activation of the NF‐kappa B pathway.^22^ Our study does not support this claim like two negative clinical trials using TNF‐α blocking drugs for the prevention of cancer‐related sarcopenia.^23^ IL‐8 can be upregulated by both IL‐1β and TNF‐α.^24^ In our study, IL‐8 contributed positively to the ‘necrotic‐anorexic’ statistical biomarker only. IL‐8 has been associated with low‐third gastric cancer especially in the presence of cachexia,^25^ and has positively correlated with TNF‐α in a population of cachectic patients with advanced prostate cancer.^26^ Our study found that these cytokines within the ‘necrotic‐anorexic’ statistical biomarker were negatively associated with the orexigenic hormone, ghrelin, as previously suggested.^27^, ^28^ Interestingly, the ‘necrotic‐anorexic’ statistical biomarker, contrary to the ‘inflammatory’ statistical biomarker, was mostly correlated with inflammation measured by CRP suggesting that IL‐8, IL‐18, MCP‐1, and/or TNFα may be responsible for increased CRP plasma levels. The most compelling finding of these analyses is that the ‘non‐anorexic/non‐inflammatory’ statistical biomarker correlated negatively with TGF‐β1 and adiponectin, and positively with leptin, which may explain its association with better appetite, higher fat mass and muscle mass, and the absence of any other CACS phenotypes.

Our finding that TRAIL contributed to the ‘non‐anorexic/non‐inflammatory’ statistical biomarker is novel. TRAIL is released by neutrophils in response to pro‐inflammatory stimuli, such as IL‐8 and TNF‐α,^29^ and can induce apoptosis in tumour cells and contribute to tumour immunosurveillance.^30^ Tumour cells, however, can be resistant to these effects.^31^ High IL‐18 and TRAIL have been suggested to be associated with apoptosis, less CACS phenotypes, and longer survival.^32^, ^33^ The present study further supports these findings. TRAIL's relationship with leptin and adiponectin remains to be explored.

How leptin and adiponectin influence CACS is not entirely clear. In healthy individuals, leptin correlates with body mass index and body fat percentage^31^ and has an anorexic effect.^34^ In patients with cancer, leptin also correlates with indices of adiposity, but unlike in healthy individuals, is positively associated with appetite.^35^, ^36^ As indicated by Wallace *et al*., low circulating leptin levels in cachectic patients are likely a consequence of decreased fat mass caused in part by anorexia.^35^ Adiponectin, on the other hand, is inversely related to body fat mass,^36^ which our results confirm.

Using RFS cluster analysis, patients were classified into four groups with specific cytokine and hormone profiles. It is not difficult to reconcile these four groups with the cachexia continuum proposed by Fearon *et al*.^7^ Group 1 could correspond to ‘no‐cachexia’; and group 2 to pre‐cachexia with a mild degree of inflammation and ‘preserved’ appetite. Group 3 could correspond to pre‐cachexia with loss of appetite; and group 4 to cachexia tending toward terminal cachexia (cytokine storm) with very high CRP. These statements could be easily debated.

Studies on treatments for patients with cancer cachexia and other chronic diseases provide evidence that the ‘inflammatory’ and ‘necrotic‐anorexic’ statistical biomarkers represent two distinct inflammatory pathways. Clinical trials of agents targeting TNF‐α in patients with cancer cachexia are not conclusive,^37^ while those targeting both IL‐6 and TNF‐α show greater promise.^38^ Furthermore, thalidomide reduces lipopolysaccharide‐stimulated release of TNF‐α, IL‐8, and IL‐18 (‘necrotic’ cytokines), but not IL‐6, IL‐10, and IL‐12 (p70) (‘inflammatory’ cytokines) from alveolar macrophages in patients with interstitial lung disease.^39^ Similarly, anti‐TNF‐α treatment downregulates IL‐18 but not IL‐12 in patients with rheumatoid arthritis.^40^

Categorizing patients into distinct groups according to their RFS could be highly relevant for cachexia research. To illustrate, administering drugs to cancer patients that focus only on inhibiting IL‐6 could be more effective when TNF‐α is low (group 3) than when it is high (groups 2 and 4). Furthermore, patients with very high IL‐6 (group 4) may never respond to such drugs. This could also be the case with ghrelin agonists, or other drugs. This methodology of classifying patients according to cytokine and hormone profiles could lead to the development of clinical trials with optimal drug combinations. Furthermore, a reduced panel of cytokines including IL‐6, IL‐18, TRAIL, and TNF‐α could be developed to produce better prognostic scores.

This study is exploratory in nature with a relatively small population size, no confirmatory cohort, a limited number of cytokines and hormones, and a wide variety of cancer types. Including additional cytokines and hormones may lead to more robust factors and/or new ones. For example, growth/differentiation factor (GDF)‐15, found to be an important cytokine for survival estimation, is highly correlated to MCP‐1,^41^ and has a similar survival predictive value as IL‐8.^3^ In future studies, GDF‐15 might be included in the ‘necrotic‐anorexic’ statistical biomarker or create a new statistical biomarker with MCP‐1 and IL‐8. In any case, as variables increase in number, it is likely that the number of statistical biomarkers identifying pathophysiological processes would also increase. We propose that the ideal way to reproduce this study would be to have specific cancer populations, not only by cancer site but also by cancer pathology, such as adenocarcinoma versus squamous cell carcinoma in lung cancer. Within the limitations of this study, we exemplified that a limited number of cytokines and hormones can be sufficient to classify patients into groups, likely or not likely, to respond to specific drug trials in the context of CACS. Therefore, it can be advantageous to consider the development of standard biomarker profiles for each cancer type to serve as inclusion/exclusion criteria for studies with the hope of improving the results of therapeutic trials. We also acknowledge that cytokines and appetite‐modifying hormones alone do not explain the complete pathogenesis of CACS. Genetics [S3], low testosterone in males [S4], and angiotensin II,^4^ among other factors, have been implicated in CACS.

## Conclusion

This exploratory study provides evidence of two distinct inflammatory pathways and one non‐inflammatory pathway with regression factor scores that characterize patients with a variety of CACS phenotypes. While independent, these pathways may be present in different profiles in cancer‐specific patient populations. These findings provide new insight about the complex pathological processes in CACS that could orient future research and the way clinical trials are approached for the palliation of this devastating syndrome.

## Conflict of interest

The authors declare no conflict of interest.

## Supporting information


**Data S1.** Supporting Information.
