# Supplementary material for: Cancer anorexia‐cachexia syndrome is characterized by more than one inflammatory pathway
Source: J Cachexia Sarcopenia Muscle. 2024 Mar 13;15(3):1041–53. doi: 10.1002/jcsm.13430 (PMC11154782; doi:10.1002/jcsm.13430)
Supplement: Supplementary file 1 — Data S1. Supporting Information. [file JCSM-15-1041-s001.pdf]

Supporting Information

Cancer anorexia-cachexia syndrome is characterized by more than one inflammatory pathway

Bruno Gagnon, Jessica Murphy, David Simonyan, Claudia A. Penafuerte, Jacinthe Sirois, Martin Chasen, Michel L. Tremblay

Contact Information:

Dr. Bruno Gagnon

Département de médecine familiale et de médecine d'urgence, Centre de recherche sur le cancer, Université Laval, Centre de recherche du CHU de Québec

9 rue McMahon, Local 1899-6

Québec, QC G1R 2J6

Table S1. Cytokine and hormone measurement parameters

**Analyte**	Company	Cat number	Kit		Sensitivity/ Limit of Detection (LOD) pg/mL	Average % CV of MFI	Assay range	%CV intra-assay	%CV inter-assay
IL-1β (pg/mL)	Biorad				0.1	3	0.2-556	≤8	≤10
IL-6 (pg/mL)	Biorad				0.1	4	5.2-18.618	≤8	≤10
IL-8 (pg/mL)	Biorad				0.04		0.4-4942.9	2.5	4.9
IL-10 (pg/mL)	Biorad				0.9	3	1.1-11.850	≤8	≤10
IL-12 (pg/mL)	Biorad				0.37	4	1.7-3.994	≤8	≤10
IL-18 (pg/mL)	Biorad				0.31		1.6-25 798	2.6	7.2
IFN-γ (pg/mL)	Biorad				0.4	4	0.7-1.814	≤8	≤10
TGF-β1 (ng/mL)	Biorad				3.9		1.69-27616	4.5	4.9
TRAIL (pg/mL)	Biorad				0.89		1.78-29188	3.2	4.5
				Specific analyte/subform	Assay sensitivity (Min detectable conc.) pg/mL		Assay range	%CV intra-assay	%CV inter-assay
Adiponectin (μg/mL)	Millipore	HADK1-61K-A	Single plex	Adiponectin (total plasma)	145.4			5.6	15
TNF-α (pg/mL)	Millipore	HMHMAG-34K	3-plex	TNF-α	0.3			3	6
Ghrelin (pg/mL)	Millipore	HMHMAG-34K	3-plex	Active ghrelin	2			2	8
Leptin (ng/mL)	Millipore	HMHMAG-34K	3-plex	Leptin	27			3	4

Table S2. Backward multivariate regression analyses of patient groups on cachexia-related outcomes

**Outcomes**	**Predictors**[a]								
Linear Regression	**Patient Groups**	*N (%)*	*B (95% CI)*		*P-value*	**Cancer Site**[b]	*B (95% CI)*		*P-value*
**z-skeletal muscle index**[c,d] (cm^2/m^2)	Group 3	28 (28.57)	-3.39 (-8.95, 2.18)	-3.56 (-7.05, -0.08)	0.045	--	--		--
	Group 4	32 (32.65)	-3.64 (-9.09, 1.81)						
	Group 2	24 (24.49)	0.06 (-5.65, 5.78)	Reference					
	Group 1	14 (14.29)	Reference						
**z-fat mass index**[e] (kg/m^2)	Group 3	28 (28.57)	-67.90 (-95.50, -40.30)	-60.24 (-83.62, -36.87)	<0.0001	--	--		--
	Group 4	32 (32.65)	-61.61 (-88.41, -34.82						
	Group 1	14 (14.29)	-41.81 (-75.17, -8.44)						
	Group 2	24 (24.49)	Reference	Reference					
**C-reactive protein** (mg/L)	Group 4	39 (29.32)	22.29 (9.17, 35.41)	14.33 (5.82, 22.84)	0.001	CRCa	21.06 (3.04, 39.07)	25.89 (13.30, 38.47)	<0.0001
	Group 3	39 (29.32)	8.19 (-4.99, 21.37)			Bca	9.72 (-9.81, 29.24)	12.58 (3.46, 21.70)	0.007
	Group 2	35 (26.32)	0.98 (-12.43, 14.38)	Reference		HBCa	6.93 (-12.20, 26.07)		
	Group 1	20 (15.04)	Reference			NSCLCa	9.42 (-9.32, 28.17)		
						PrCa	7.65 (-16.73, 32.02)		
						ENT	-0.15 (-17.96, 17.67)	Reference	
						PaCa	-5.87 (-22.11, 10.36)		
						UGICa	Reference		

Logistic Regression	**Patient Groups**	*N (%)*	*OR (95% CI)*		*P-value*				
**Cachexia**[f,g] (Yes versus No)									
	Group 4	39 (29.32)	10.96 (2.97, 49.49)	4.01 (1.84, 9.09)	0.0006	CrCa	3.77 (0.67, 23.68)	2.91 (1.36, 6.41)	0.007
	Group 3	39 (29.32)	5.19 (1.43, 22.72)			PaCa	3.25 (0.69, 16.74)		
	Group 2	35 (26.32)	1.98 (0.48, 9.18)	Reference		Bca	1.15 (0.17, 7.85)		
	Group 1	20 (15.04)	Reference			HBCa	1.19 (0.18, 7.85)		
						NSCLCa	1.06 (0.17, 6.75)	Reference	
						ENT	0.81 (0.14, 4.69)		
						UGICa	Reference		
Cumulative Ordinal Regression	**Patient Groups**	*N (%)*	*OR (95% CI)*		*P-value*				
**6-Month recall weight loss** (%) (< 2; 2-5; ≥ 5)	Group 4	39 (29.32)	5.63 (1.95, 17.37)	4.23 (1.94, 9.78)	0.0003				
	Group 3	39 (29.32)	2.05 (0.75, 5.84)			--	--		--
	Group 2	35 (26.32)	0.99 (0.35, 2.85)	Reference					
	Group 1	20 (15.04)	Reference						
**Body mass index** (kg/m^2) ≥ 30.0; 25.0 – 29.9;20.0 – 24.9; < 20.0	Group 3	39 (29.32)	6.50 (2.55, 17.15)						
	Group 4	39 (29.32)	5.59 (2.24, 14.46)	5.54 (2.48, 12.84)	<0.0001	--	--		--
	Group 1	20 (15.04)	4.10 (1.39, 12.48)						
	Group 2	35 (26.32)	Reference	Reference					

[a] All initial models included group, sex, age, cancer site, and cancer stage. Predictors with $P < 0.05$ were subsequently removed in the backward regression. The results for the final model are presented.

[b] Cancer sites: BCa: Breast cancer; CRCa: Colorectal cancer; ENT: Ear, nose, throat cancer; HBCa: Hepatobiliary cancer; NSCLCa: Non-small-cell lung cancer; PaCa: Pancreatic cancer; PrCa: Prostate cancer; UGICa: Upper gastrointestinal cancer

[c] z-skeletal muscle index: sex-standardized skeletal muscle index based on cut-points of < 55 cm^2/m^2 for men and < 39 cm^2/m^2 for women [9]

[d] Sex (Reference=male): B (95% CI) = 4.74 (1.28, 8.19); $P = 0.008$

[e] z-fat mass index: sex-standardized fat mass index [13]

[f] Defined according to the International Consensus Statement: 6-month recall weight loss > 5%, or a 6-month recall weight loss 2-5% plus sarcopenia and/or a BMI < 20 kg/m^2 [10]

[g] Patients with prostate (PrCa) cancer (n=6) were excluded from the analysis because they all had cachexia, causing convergence failure of the logistic regression model.

} Predictors with similar regression coefficients were combined to improve power.

Figure S1. Kaplan-Meier curves according to patient groups

+ censored

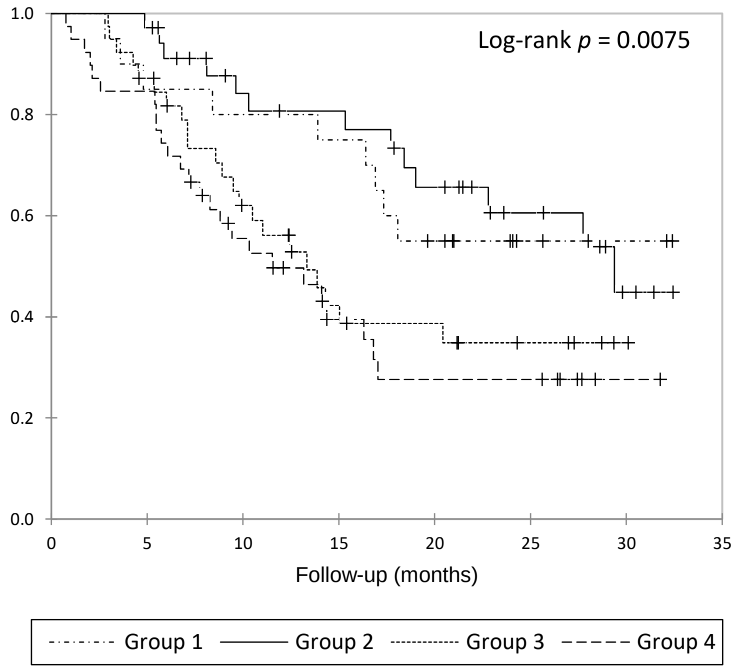

Group	Subjects	Events	Censored	Median Survival (95% CI) (months)
1	20	9	11	22.0 (13.90, -)
2	35	13	22	29.37 (18.40, -)
3	39	22	17	13.33 (9.50, -)
4	39	25	14	11.53 (7.17, 16.80)

Supplementary References

[S1] Ribaudo JM, Cella D, Hahn EA, Lloyd SR, Tchekmedyian NS, Von Roenn J, et al. Re-validation and shortening of the Functional Assessment of Anorexia/Cachexia Therapy (FAACT) questionnaire. Qual Life Res Int J Qual Life Asp Treat Care Rehabil 2000;9:1137–46.

[S2] Podsiadlo D, Richardson S. The Timed "Up & Go": A Test of Basic Functional Mobility for Frail Elderly Persons. J Am Geriatr Soc 1991;39:142–8.

[S3] Johns N, Stretch C, Tan BHL, Solheim TS, Sørhaug S, Stephens NA, et al. New genetic signatures associated with cancer cachexia as defined by low skeletal muscle index and weight loss. J Cachexia Sarcopenia Muscle 2017;8:122–30.

[S4] Burney BO, Hayes TG, Smiechowska J, Cardwell G, Papusha V, Bhargava P, et al. Low Testosterone Levels and Increased Inflammatory Markers in Patients with Cancer and Relationship with Cachexia. J Clin Endocrinol Metab 2012;97:E700–9.
